# Supplementary material for: Hypericin Suppresses SARS-CoV-2 Replication and Synergizes with Antivirals via Dual Targeting of RdRp and 3CLpro
Source: Microorganisms. 2025 Apr 27;13(5):1004. doi: 10.3390/microorganisms13051004 (PMC12114490; doi:10.3390/microorganisms13051004)
Supplement: Supplementary file 1 [file microorganisms-13-01004-s001.zip › microorganisms-3499218-supplementary.pdf]

SUPPLEMENTAL MATERIAL

Methodology

SARS-CoV-2 3Cl (Mpro) and RdRp initial structures

This experiment used the most promising results obtained by our group in previous docking experiments for SARS-CoV-2 3Cl and RdRp in a complex with HY [39]. The complexes are formed by the protein structure of SARS-CoV-2 3Cl (Mpro) (PDB: 6LU7) [46] and the SARS-CoV-2 RdRp domain (7bv2) [47]. The protein structures were retrieved from the PDB database, and missing regions were modeled using AutoModel [48] via homology modeling. To enhance the structural quality and packing of the structural model of RdRp, the first four N-terminal residues were removed. This impacts all the analyses in our simulation experiments as the obtained residue numbers are different (-4) if compared with the database sequences. For both protein structures, all water molecules and heteroatoms were excluded to optimize the conditions for docking experiments. In both prior docking studies and the current simulation experiments, Mg^{2+} ions were retained within the active site of SARS-CoV-2 RdRp, ensuring an accurate representation of the enzyme's catalytic environment.

Molecular simulation of the RdRp and 3CL (Mpro) in complex with HY

Starting from the protein-ligand complex with the best interaction energy obtained in previous docking experiments [39], molecular simulations were performed to define the stability of the ligand positioning in the complex and the definition of the protein residues essential for maintaining the ligand binding.

The RdRp-HY complex is composed of 1120 amino acids (911 from chain A, 114 from chain B, 67 from chain C and 28 from chain D), a HY ligand (Hyp), two Ca2+ ions, and three Zn2+ ions (two of them essential cofactors for the polymerization reaction catalyzed by RdRp) was initially placed in a triclinic simulation box (4.05 x 4.3 x 2.8 nm) with periodic conditions. Approximately 38,260 explicit water molecules and 53 Na+ and Cl- ions were added to simulate the physiological conditions of 150 mM salt. The final simulation system contains 132,812 atoms.

Following the same steps, the final simulation 3Cl (Mpro)-HY system contains 35.064 atoms. The initial complex, composed of 306 residues and a HY ligand, was accommodated in a triclinic simulation box (53.25 x 67.86 x 87.37) with 10.090 water molecules and 28 Na+ and Cl- ions (150 mM salt). The obtained systems were parameterized using the OPLS-2005 force field and SPCE water model [49] in Desmond-GPU-Maestro 2022-4 [50]. The simulation system was equilibrated for five ns in a mixed NVT/NPT system [51].

Three to five replicates were produced for data collection. Each replicate run goes through several stages according to the Maestro instructions as follows:

1) Brownian dynamics of the NVT ensemble: temperature of 10 K, small time steps, restraints on heavy solute atoms for 100 ps.
2) Berendsen dynamics of the NVT ensemble: temperature of 10 K, small time steps, constraints on heavy solute atoms, and 12 ps.
3) Berendsen dynamics of the NPT ensemble: temperature of 10 K, constraints on heavy solute atoms, and 12 ps.
4) Berendsen dynamics of the NPT ensemble: temperature of 310 K, constraints on heavy solute atoms, and 12 ps.
5) Berendsen dynamics of the NPT ensemble: temperature of 310 K, no constraints, and 500 ps.
6) Data production: Berendsen dynamics of the NPT ensemble is performed at 310 K without constraints until the desired simulation time.

Data production simulations were performed on a Linux server at LQFPP-UENF for 50 to 200 ns using an NPT system at 300 K and 1 bar of pressure using a variant of the Nosé-Hoover thermostat [52] and the Martyna-Tobias-Klein algorithm as barostat [53]. The simulation time step used was 2.0 fs in the RESPA integration method, and the electrostatic forces were calculated using the u-series method (10) with a cutoff of 0.9 nm. The experiments were performed in triplicate, and the interaction data were obtained from Maestro's "Simulation Interaction Diagram."

The protein-ligand interactions were monitored throughout the simulation. These interactions are categorized into four types: hydrogen bonds, hydrophobic, ionic, and water bridges. Each interaction type contains more specific subtypes, explored using the 'Simulation Interaction Diagram' panel. The stacked bar graphs were normalized along the trajectory; for example, a value of 0.7 suggests that the specific interaction was maintained for 70% of the simulation time. Values above 1.0 are possible because some protein residues can make multiple contacts with the ligand as follows:

1- Hydrogen bonds (H-bonds): Are significant in ligand binding. Hydrogen bonds between a protein and a ligand can be divided into four subtypes: backbone acceptor, backbone donor, side chain acceptor, and side chain donor. The current geometric criteria for protein-ligand H-bonding are a distance of 2.5 Å between the donor and acceptor atoms (D—H···A); a donor angle of ≥120° between the donor-acceptor hydrogen atoms (D—H···A); and an acceptor angle of ≥90° between the atoms of the atom bound to the hydrogen acceptor (H···A—X).

2- Hydrophobic contacts: These are divided into three subtypes: π-Cation, π-π, and other nonspecific interactions. Generally, these interactions involve a hydrophobic amino acid and an aromatic or aliphatic group in the ligand, but we also include π-Cation interactions. The current geometric criterion for hydrophobic interactions is as follows: π-Cation — Aromatic and charged groups within 4.5 Å; π-π — Two aromatic groups stacked face-to-face or face-to-edge; Other — A nonspecific hydrophobic side chain within 3.6 Å of the aromatic or aliphatic carbons of a ligand.

3- Ionic or polar interactions: These are between oppositely charged atoms within 4 Å of each other and do not involve a hydrogen bond. We also monitor Protein-Metal-Ligand interactions, defined by a coordinated metal ion within 4 Å of the heavy atoms of the protein and ligand (except carbon). All ionic interactions are divided into two subtypes: those mediated by a protein backbone or side chains.

4- Water bridges: A water molecule mediates hydrogen-bonded protein-ligand interactions. The geometry of the hydrogen bond is slightly relaxed from the standard definition of an H-bond. The current geometric criteria for a protein-water or water-ligand H-bond are: a distance of 2.8 Å between the donor and acceptor atoms (D—H···A); a donor angle of ≥110° between the donor-hydrogen-acceptor atoms (D—H···A); and a donor angle of ≥90° between the atoms of the atom bonded to the hydrogen acceptor (H···A—X).

Metadynamics experiments

Metadynamics experiments were performed using Desmond-GPU-Maestro [50] to estimate the interaction energy between HY and SARS-CoV-2 3Cl (Mpro) and RdRp. After equilibration for 10 ns in explicit water and 150 mM salts, the systems described above were subjected to metadynamics using the dedicated panel in Maestro ((https://support.schrodinger.com/s/article/1621, accessed on 03/2023). As described above, the already solvated complex was loaded into the Metadynamics panel to prepare the input files for metadynamics. The energy variations of the complex were collected due to the variation of two collective variables (CV), the distances between the centers of mass of the protein residues and the ligand.

For RdRp-HY complex, the CV distance was defined between the center of mass of residues Arg552 (CV1) and Lys846 (CV2) to the center of mass of HY, with values of 2.6 and 3.6 nm, respectively, as the maximum collection wall. For the 3Cl-HY complex, the CV of distance was defined between the center of the mass of HY and the center of the mass of CYs38 (CV1) and Thr26 (CV2) with the collection wall values of 2.0 and 1.6, respectively.

The Gaussian width and height in metadynamics experiments were defined as 0.05 Å and 0.01 kcal/mol, respectively. The wall at the defined CV value allows the ligand to float, exploring different binding sites on the protein surface and being more or less subjected to contact with the solvent. However, it prevents it from moving too far from the studied binding site. The simulation temperature and pressure were defined as described above for the other simulations. The Gaussian injection time interval was set at 0.09 ps. Three replicates were collected, as described in the previous simulations. However, in the data production, the Martyna-Tobias-Klein dynamics [52] of the NPT ensemble with metadynamics is performed at 310 K without restrictions for 50 to 100 ns. The variables of each experiment were collected and plotted in a two-dimensional graph in Maestro to define wells of best free energy of the complex (ΔG).

The structures with the lowest ΔG value were analyzed, and the observed protein-ligand contacts were compared to those obtained in previous docking experiments and molecular simulations.

RESULTS

To deepen the molecular docking studies previously presented during the pandemic that point to HY as a possible virucidal agent, molecular simulation studies were further developed using the best complexes obtained in the docking experiments as star points [39]. Continuing the line of the initial hypothesis of simultaneous interaction of HY with both proteins and seeking to elucidate and explicitly look for the molecular structures of the recognition and intended interaction of 3Cl (Mpro) and RdRp with Hy, we carried out molecular simulations and metadynamics experiments that we will delve deeper into in this part of the document.

Molecular simulations and metadynamics of SARS-CoV-2 3CL (Mpro) in complex with HY.

During the simulations, the ligand did not show large movements. Relatively low R.M.S.D. values were observed for the ligand in all replicates (Figure S1: A). This suggests that the positioning obtained in the docking experiments points to an energetically favorable positioning (Figure 5: A). HY continues to block the active site of 3Cl, partially inserting itself into the recognition space of possible substrates and presenting itself through its structural positioning in the complex as a competitive inhibitor (Figure 5: B).

It also was observed that HY maintains a pattern of interaction with 3Cl of hydrogen bonds mediated by residues Asp187, Arg188, Gln192, and Thr190, aided by hydrophobic interactions with Met49 on one side of the structure and water bridges with Glu166 on the opposite side (Figure S1: B-D and Figure 5: C). The interacting residues maintain the contacts over most of the simulation over all the analyzed replicas (Figure S1:B-D), and no significant rearrangements were observed in the analyzed trajectories. Other contacts were observed at minor levels or only in one of the replicas and were considered of minor importance for recognition and complex formation. Only contacts lasting more than half of the trajectories were deemed essential for the interaction (Figure S1: C). During its interaction with the enzyme, HY remains partially inserted in the enzyme's active site, on the run as much as possible from the water phase, but still partially in contact with water (Figure 5: E).

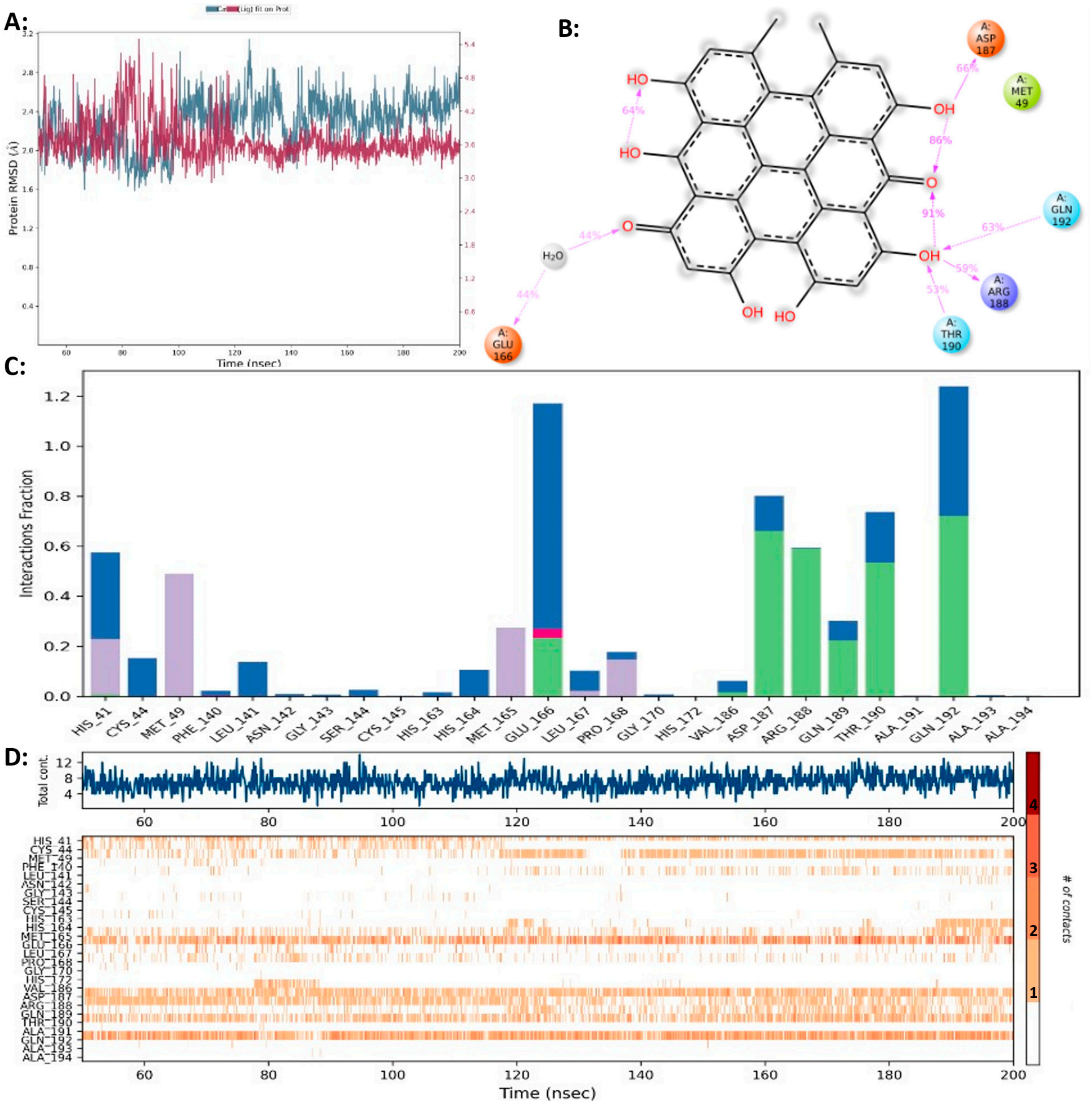

Figure S1: Simulation of 3CL_Hy complex for 200ns. The first 50 ns were used for complex equilibrium, and the last 150 for production simulation. A: Calculated RMSD for the protein backbone (in gray) and HY (in red) over the simulation. B: Most important protein residues contact HY during the production simulation. Electrostatic contributions in red (negative) and blue (positive). Hydrophobic contributions represented in green and hydrogen bonds in light blue. C: Fraction over the simulation trajectory for residues contacting HY. D: Representation of the contacts over the simulation time (50 to 200 ns).

The metadynamics experiments used the same system as the molecular dynamics experiments, but the equilibrium time was reduced to 10 ns. The rationale of the metadynamics experiment, different from the linear collection of the previous molecular simulation, uses an adaptive method that forces the system outside its energy minimum. During the simulation, we collected two variables (CV) for the distance between the centers of mass of HY, Cys38, and Thr26 to analyze the affinity energy in the complex. The result in all replicates indicates a single pool of the lowest free energy with an estimated value of -15 to -17 kcal/mol (Figure 5: D), which represents the accommodation of HY blocking the enzyme's active site

and in contact with the identical residues already identified by the molecular simulation (Figure 5: C and Figure S1: B-D). The ligand performs several hydrogen bonds with residues from Gln192 to Asp 187, helped by the accommodation of hydrophobic character mediated by Met49 and Met165 (Figure 5: E). The convergence of the HY binding site highlighted by the two different experiments, in addition to showing a large number of identical residues critical for contacts, strongly suggests that HY fits into the active site of 3CL (Mpro) and behaves as a competitive inhibitor of this enzyme. This fact may imply an essential contribution to its virucidal activity.

Molecular simulations and metadynamics of SARS-CoV-2 RdRp in complex with HY.

Minor accommodation of HY at the binding site was observed in all simulations of the RdRp-HY complex obtained in previously published docking experiments [39]. However, it demonstrated a discrete displacement and low RMSD (Figure S2: A). Positioned very close to the Mg ions in the catalytic site of RdRp and occupying part of the interaction site with RNA, HY sits on top of Trp797, maintaining Pi stacking between its aromatic structures (Figure 2: B-C). This positioning is supported by hydrogen bonds or water bridges from residues in the Asp801 to Glu808 loop and water bridges with Glu799. On the opposite side of HY, we still have essential hydrogen bonds and water bridges with Ser604 and Asp605 (Figure S2: B-D and Figure 6: C and E). In all replicas, only contacts lasting more than half of the trajectories were considered fundamental for the RdRp-HY interaction (Figure S2: C-D). During the HY interaction with the enzyme, the inhibitor remains inserted in the enzyme's hydrophobic cleft near the active site (Figure 6: B-C), again as much as possible from the water phase.

At the same time, during the metadynamics experiments, we collected two variables (CV) for the distance between the centers of mass of HY, Lys157, and Arg552 to analyze the affinity energy in the complex when moving the ligand in the x-y space in the active site. The result indicates a single pool with free energy estimated in -12 to -13 kcal/mol (Figure 1: D), which represents the hydrophobic interaction of HY in the enzyme cleft near the active site. Again, as in the HY-3Cl experiments, both simulation and metadynamics experiments pointed to the identical residues essential for the complex formation (Figure 1: C and Figure S1: B-D).

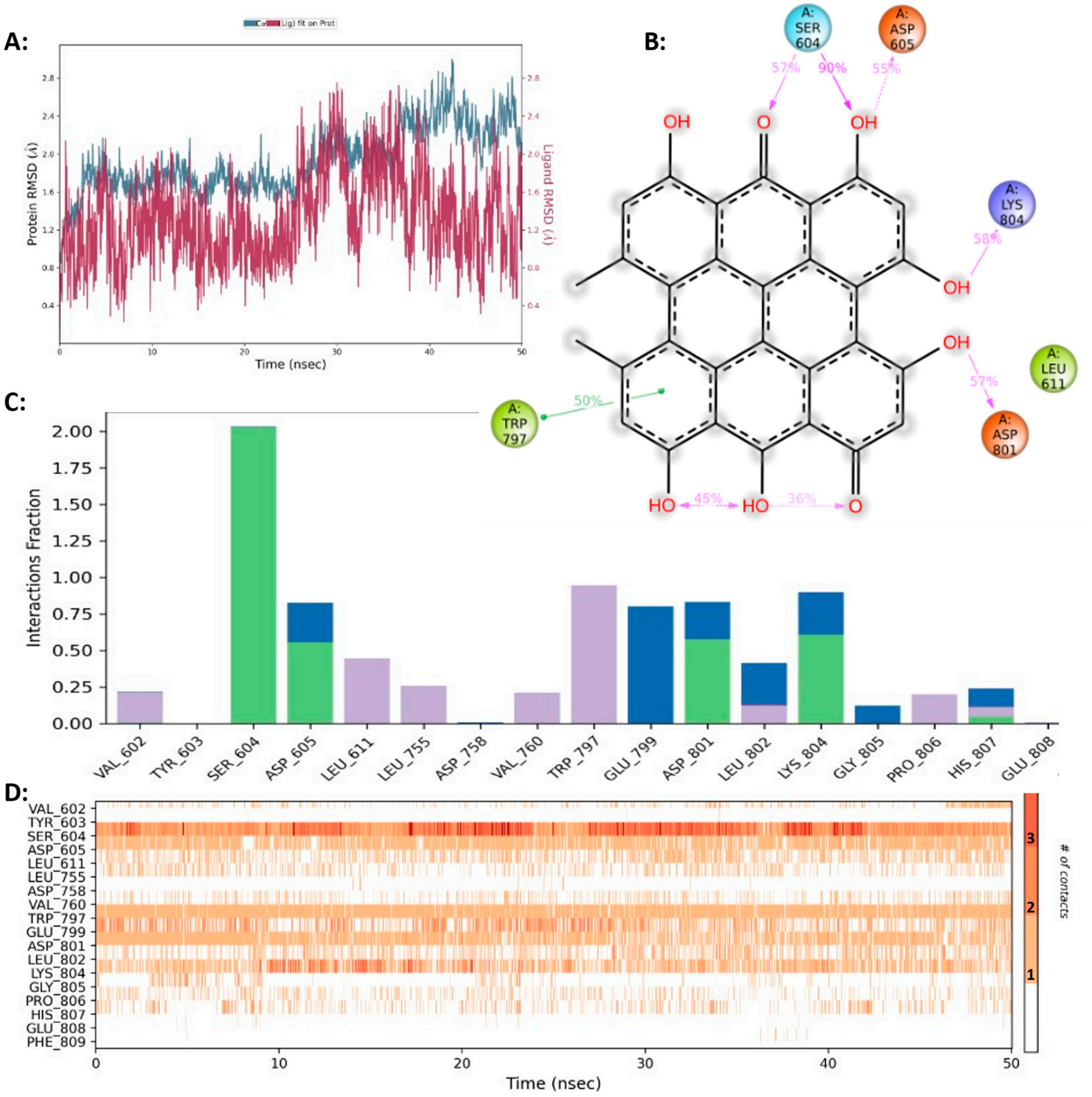

Figure S2: Simulation of RdRp_Hy complex for 50ns. A: Calculated RMSD for the protein backbone (in gray) and HY (in red) over the simulation. B: Most important protein residues contacting HY over the simulation. Electrostatic contributions in red (negative) and blue (positive). Hydrophobic contributions represented in green and hydrogen bonds in light blue. C: Fraction over the simulation trajectory for residues contacting HY. D: Representation of the contacts over the simulation time (50ns).

HY-contacting residues in both complexes are conserved in the Coronaviridae family.

After identifying the critical residues involved in forming HY inhibitory complexes with the SARS-CoV-2 enzymes 3CL and RdRp, it is essential to assess the conservation of these residues across different types and variants of coronaviruses. To achieve this, we employed the sequences of 3CL (chain A from PDB 6LU7, (2697049) and RdRp (chain A from PDB 7BV2, (2697049) in a BLASTp search against the SeqRef database, restricted to Coronaviridae sequences (taxid: 11118). Multiple sequence alignments were generated for each protein (Figure S3).

For 3CL, the critical residues Asp187, Arg188, Gln192, Glu166, and Thr190 (Mpro; Figure S3: A) were found to be highly conserved across a wide range of coronaviruses infecting various hosts. Similarly, in RdRp, the loop residues Asp801-Glu808 and Glu799, Ser604, and Asp605 (Figure S3: B) exhibited high conservation.

The observation that these residues, crucial for HY recognition in both enzymes, are remarkably conserved across coronaviruses suggests that HY could potentially exhibit efficacy against a broad spectrum of SARS-CoV-2 variants, including those currently in circulation and future modifications.

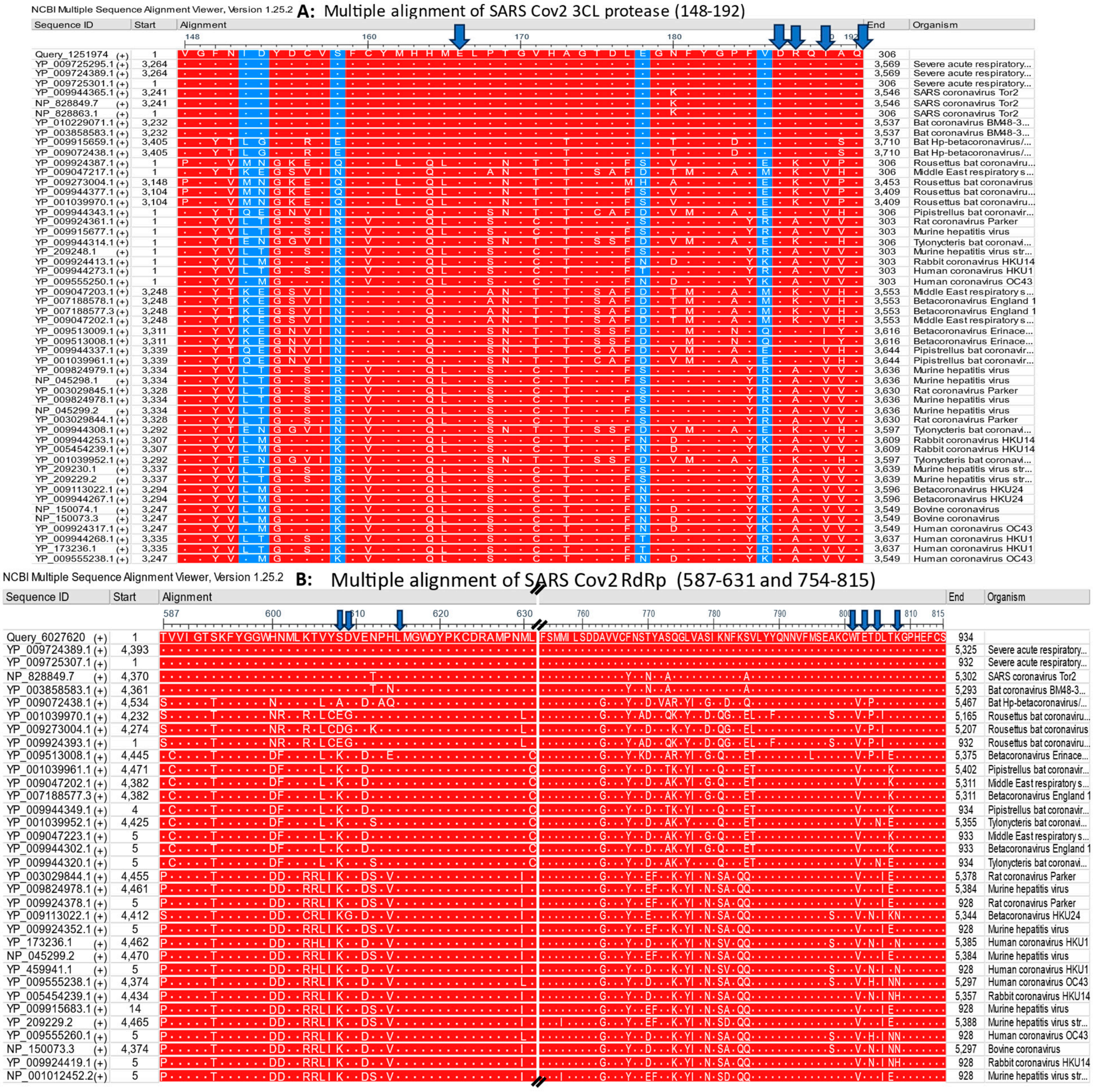

Figure S3: Multiple alignments of SARS-Cov-2 3CL (Mpro) and RdRp protein sequences obtained in the NCBI MSA Viewer. A: Using the blastp algorithm, we searched the FASTA format of the 302 residues from chain A of 6LU7 pdb structure against the RefSeq database limited to records that include Coronaviridae (taxid:11118). B: The same blastp algorithm was used to search in the RefSeq database limited to records that include Coronaviridae (taxid:11118) for similar protein sequences for the 941 RdRp residue from chain A in the 7BV2 pdb structure. Identical residues are represented in the alignment as dots. Discussed positions are pointed out with blue arrows. Colored in red are the most conserved residues in the alignment.
